# Preoperative vitamin D deficiency and postoperative hypocalcemia in thyroid cancer patients undergoing total thyroidectomy plus central compartment neck dissection

**DOI:** 10.18632/oncotarget.17690

**Published:** 2017-05-08

**Authors:** Xiaofei Wang, Jingqiang Zhu, Feng Liu, Yanping Gong, Zhihui Li

**Affiliations:** ^1^ Department of Thyroid and Breast Surgery, West China Hospital, Sichuan University, Chengdu, China; ^2^ Department of Head and Neck Surgery, Sichuan Cancer Hospital & Institute, Chengdu, China

**Keywords:** vitamin D deficiency, hypocalcemia, thyroid cancer, total thyroidectomy

## Abstract

**Background:**

There appears to be a lack of consensus whether preoperative vitamin D deficiency (VDD) increases the risk of postoperative hypocalcemia and decreases the accuracy of postoperative parathyroid hormone (PTH) in predicting hypocalcemia in thyroid cancer patients undergoing total thyroidectomy (TT) plus central compartment neck dissection (CCND). This study aims to address these issues.

**Method:**

All consecutive thyroid cancer patients who underwent TT plus CCND were retrospectively reviewed through a prospectively collected database between October 2015 and April 2016 in a tertiary referral hospital. The multivariate analysis was performed to identify the significant predictors for hypocalcemia. Receiver operator characteristic curve (ROC) was created and the area under the ROC was used to evaluate the predictive accuracy of postoperative PTH and compared between patients with or without VDD.

**Results:**

A total of 186 patients were included. The incidence of VDD was 73.7% (137 patients). The incidence of biochemical and symptomatic hypocalcemia was similar in patients with or without VDD (*P* = 0.304 and 0.657, respectively). Multivariate analysis showed that only postoperative PTH was an independent predictor of symptomatic hypocalcemia (OR = 8.05, 95%CI = 3.99-16.22; *P* = 0.000). The area under the ROC was similar between patients with preoperative vitamin D level < 20 and ≥20 ng/mL (0.809 versus 0.845, *P* = 0.592).

**Conclusion:**

VDD was not a significant risk factor for hypocalcemia following TT+CCND, and did not affect the accuracy of postoperative PTH as a predictor of postoperative hypocalcemia. Thus, routine preoperative screening for vitamin D seems to be unnecessary.

## INTRODUCTION

Transient hypocalcemia is the most common complication of total thyroidectomy (TT) [1, 2], which has been reported to occur in up to 54% of patients [3]. It is a challenge for thyroid surgeons since it often leads to increased biochemical test frequency and prolonged hospitalization time [4, 5]. Although higher incidence has been reported by previous literature, causes and mechanisms of post-thyroidectomy hypocalcemia remain unclear. Many factors have been postulated to increase the risk of hypocalcemia following TT, including surgical trauma, devascularization, or inadvertent removal of the parathyroid glands, surgeon volume, extent of cervical lymph node dissection, and so on [6–10]. Recently, Vitamin D deficiency (VDD) has also been recognized as a risk factor for postoperative hypocalcemia following TT in patients with benign thyroid diseases (nontoxic multinodular goiter or Graves’ disease) [11–14]. However, the evidence is scarce and somewhat inconsistent regarding this association in thyroid cancer patients [15–17]. Different from patients with benign thyroid diseases, the majority of thyroid cancer patients are usually underwent central compartment neck dissection (CCND) besides TT in China. These patients might more often experience postoperative hypocalcaemia.

Symptoms of hypocalcemia usually occur 24-48 hours after surgery, so accurate prediction has potential influence on management strategies and shorten hospital stay. Early postoperative serum parathyroid hormone (PTH) level has been considered to be an accurate marker for predicting the development of postoperative hypocalcemia [2, 3, 6]. However, preoperative VDD might lead to secondary hyperparathyroidism and an increased bone turnover, subsequently leading to an increased PTH level [18, 19], which would affect the accuracy of postoperative PTH in predicting postoperative hypocalcemia [19, 20], but other studies didn't confirm this influence [15, 16].

Therefore, in this study, we retrospectively analyzed our perspectively collected data to further clarify the association between preoperative vitamin D levels and postoperative hypocalcemia in thyroid cancer patients undergoing TT and CCND, a sub-population at high risk of developing hypocalcemia [9, 21, 22], which was a good model to analysis hypocalcemia risk. Furthermore, we also investigated whether preoperative vitamin D levels would affect the accuracy of postoperative PTH in predicting post-thyroidectomy hypocalcemia.

## RESULTS

A total of 186 patients were included in this study. Among them, 140 (75.3%) were female and 46 (24.7%) male. The mean age of patients was 42.98 ± 13.20 years (range, 18-76 years). Of the 186 patients, 125 (67.2%) cases underwent bilateral CCND, 68 (36.6%) underwent MRND, and 64 (35.0%) underwent parathyroid glands autotransplantation. The mean vitamin D level of the entire cohort was 16.8 ± 6.89 ng/mL. Of the patients, 23 (12.4%) had serum 25OHD levels of <10 ng/mL, 114 (61.3%) had serum 25OHD levels of 10–20 ng/mL, and 49 (26.3%) had serum 25OHD levels of >20 ng/mL. The incidence of laboratory and symptomatic hypocalcemia was 53.2% (99 patients) and 38.2% (71 patients), respectively. The serum calcium levels returned to normal in all patients with hypocalcemia and the symptoms of hypocalcemia resolved in all patients with symptomatic hypocalcemia within one month after surgery. Only one patient had persistent symptoms of hypocalcemia with below normal PTH levels at six months after operation. This patient has a normal level of preoperative serum vitamin D (66.4 nmol/L).

The demographic and clinicopathological characteristics of patients in VDD and VDS groups were presented in Table 1. There was no significant difference between the groups regarding all the parameters, except the level of preoperative 25OHD (mean: 13.46 ng/mL vs. 26.12 ng/mL, *P* = 0.000). The incidence of biochemical hypocalcemia and symptomatic hypocalcemia was similar in two groups (55.5% vs. 46.9%, *P* = 0.304 and 37.2% vs. 40.8%, *P* = 0.657, respectively). There were 23 patients with severely VDD (< 10 ng/mL). Among them, the incidence of biochemical hypocalcemia and symptomatic hypocalcemia was 60.9% (14/23) and 47.8% (11/23), which was not different from that of patients with 25OHD level > 10 ng/mL (*P* = 0.433 and 0.309, respectively).

**Table 1:** The main characteristics of patients with vitamin D deficiency and sufficiency

Variables	VDD (n=137)	VDS (n=49)	*P* Value
Age (years)	42.0±12.8	45.7±14.1	0.099
Gender (female/male)	102/35	38/11	0.666
Tumor size (cm)	1.9±0.34	1.8±0.75	0.530
Concomitant hashimoto's thyroiditis (%)	22 (16.1)	5 (10.2)	0.318
Bilaterality (%)	35(25.5)	16(32.7)	0.339
Extrathyroid extension (%)	92(67.2)	29(59.2)	0.315
No. of identified PTG			
4/3/2 PTG	126/9/2	45/2/2	0.491
No. of PTG autotransplantation			
0/1/2 PTG	89/41/7	33/15/1	0.620
Extent of CCND			
Unilateral/Bilateral	47/90	14/35	0.463
MRND			
No/Unilateral/ Bilateral	87/38/12	31/15/3	0.805
Stage			
T1a/T1b/T2/T3/T4a/T4b	23/8/2/97/5/2	7/6/2/31/2/1	0.442
N0/N1a/N1b	62/31/44	26/11/12	0.559
Preoperative serum Ca (mmol/L)	2.28±0.07	2.32±0.15	0.903
Preoperative serum VD (ng/mL)	13.46±3.59	26.12±5.11	**0.000**
Preoperative serum PTH (pmol/L)	5.78±1.90	5.23±1.59	0.072
Postoperative serum PTH (pmol/L)	2.95±1.89	2.83±1.88	0.710
Postoperative serum Ca (mmol/L)	1.95±0.17	1.97±0.17	0.347
Biochemical hypocalcemia (%)	76(55.5)	23(46.9)	0.304
Symptomatic hypocalcemia (%)	51(37.2)	20(40.8)	0.657

Abbreviations: VDD: Vitamin D deficiency; VDS: Vitamin D sufficiency; PTG, Parathyroid gland; CCND, Central compartment neck dissection; MRND: modified radical neck dissection; Ca: Calcium; VD: Vitamin D; PTH: Parathyroid hormone. Serum factors were presented by mean ± standard deviations

Multiple logistic regression analysis showed that only postoperative PTH was an independent predictor of symptomatic hypocalcemia (OR = 8.05, 95%CI = 3.99-16.22; *P* = 0.000). However, preoperative vitamin D level (using the cutoff value of 10 or 20 ng/mL), parathyroid gland autotransplantation and extent of CCND were not associated with the development of symptomatic hypocalcemia (Table 2).

**Table 2:** Linear logistic regression analysis between symptomatic hypocalcemia and independent parameters

Parameters	Odds ratio (95% CI)	*P* value
**Postoperative PTH < 1.6 pmol/L**	8.05 (3.99-16.22)	**0.000**
**Preoperative 25OHD <10 ng/mL**	1.01 (0.34-2.98)	0.992
**Preoperative 25OHD <20 ng/mL**	1.12 (0.40-1.95)	0.886
**Parathyroid gland autotransplantation**	1.94 (0.94-4.01)	0.073
**Bilateral CCND**	1.24 (0.59-2.60)	0.575

Abbreviations: PTH, parathyroid hormone; 25OHD, 25-hydroxyvitamin D; CCND, central compartment neck dissection.

Table 3 compared the preoperative and postoperative PTH levels according to the vitamin D status and presence or absence of symptomatic hypocalcemia. A significant relationship was observed between postoperative PTH and hypocalcemia in patients with vitamin D deficiency or sufficiency (both *P* = 0.000). There was also a significant difference in the percentage of postoperative PTH less than 1.6 pmol/L in patients with or without symptomatic hypocalcemia in both the groups (*P* = 0.000 and 0.001, respectively).

**Table 3:** Comparison of the preoperative and postoperative PTH levels according to their vitamin D level and presence or absence of symptomatic hypocalcemia

	25OHD <20 ng/mL			25OHD ≥20 ng/mL		
	Symptomatic hypocalcemia		*P* value	Symptomatic hypocalcemia		*P* value
	Presence(n=51)	Absence(n=86)		Presence(n=20)	Absence(n=29)	
**Preoperative PTH**	5.9±1.86	5.7±1.92	0.453	5.2±1.92	5.2±1.35	0.966
**Postoperative PTH**	1.7±1.29	3.7±1.81	0.000	1.6±1.43	3.7±1.73	0.000
**Postoperative PTH**	32 (62.7)	14 (16.3)	0.000	13 (65.0)	5 (17.2)	0.001
**< 1.6 pmol/L n (%)**						

Abbreviations: 25OHD, 25-hydroxyvitamin D; PTH, parathyroid hormone.

**Figure 1:**
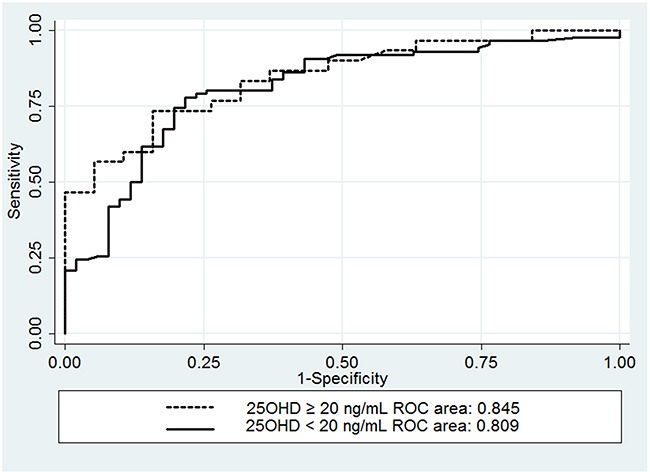
Receiver operator characteristic curves comparing the accuracy of serum PTH in predicting postthyroidectomy hypocalcemia in patients with a serum vitamin D concentration ≥20 ng/mL and those with a serum vitamin D concentration <20 ng/mL

The ROC curves for postoperative PTH were created for both groups to assess the accuracy of PTH as a predictor for the development of post-TT hypocalcemia (Figure 1). The area under the ROC curves for patients with VDD and VDS were 0.809 and 0.845, respectively (*P* = 0.592).

## DISCUSSION

VDD has a high prevalence in many countries, which has a significant burden on public health owing to its association with bone disease, cancer, lipid metabolism defects, diabetes, and heart disease [23–25]. However, there is no consensus about the accurate definition of VDD. Although VDD is defined as a serum 25OHD level less than 10 ng/mL and VDI is defined as less than 20 ng/mL by WHO criteria, most experts suggested it is more appropriate to set the cutoff at 20 ng/mL and 30 ng/mL for VDD and VDI, because serum 25OHD level is inversely associated with serum PTH until the former reached to 30 ng/mL [24, 26, 27]. Therefore, we adopted the cutoff of 20 ng/mL for VDD in the present study. According to this criterion, there was a high prevalence of VDD (73.7%) in our cohort.

Vitamin D plays a critical role in calcium homeostasis by directly regulating the intestinal calcium absorption and indirectly regulating parathyroid hormone (PTH) secretion via its effects on serum calcium. Therefore, preoperative vitamin D level might affect postoperative serum calcium and PTH metabolism. When patients have impaired parathyroid function, sufficient vitamin D could promote the intestinal calcium absorption to maintain calcium homeostasis. However, this regulatory mechanism of increased calcium absorption is damaged in the case of VDD [13, 28]. Therefore, it seems to be reasonable that preoperative VDD should increase the incidence of postoperative hypocalcemia in patients undergoing TT+CCND, which usually suffered hypoparathyroidism because of parathyroid ischemia/injury or inadvertent resection.

Very few studies have investigated the influence of VDD on postoperative hypocalcemia after TT+CCND with inconsistent results to date. Griffin et al [29] and Cherian et al [16] reported that VDD had no significant effect on the risk of postoperative hypocalcemia in patients underwent TT. However, both studies included heterogeneous population with a large number of patients with benign thyroid diseases and only a small number of patients undergoing concomitant CCND. Lee et al [30] reported that preoperative vitamin D level was not a predictor of hypocalcemia among a series of 134 patients with thyroid cancer underwent TT+CCND, whereas Kim et al [17] reported that VDD was a significantly factor for increased postoperative symptomatic hypocalcemia in a series of 267 patients undergoing TT plus CCND.

In the present study, to reduce potential confounders influence, we analyzed the data from a similar population with same pathology and similar surgery extent performed by an experienced thyroid surgeon. The result suggested that there was no significant association between preoperative serum vitamin D level and postoperative hypocalcemia in thyroid cancer patients underwent TT+CCND. This finding is consistent with that of Lee et al [30] but contrary to that of Kim et al [17]. Even if we defined VDD as vitamin D levels less than 10 ng/mL, which was the cutoff value used by Kim et al [17], the result is still similar. One explanation for our findings may be due to the fact that 1,25 dihydroxyvitamin D, converted from 25OHD, is the biologically active form of vitamin D, which is tightly regulated by PTH; post-thyroidectomy hypoparathyroidism results in decreased conversion of 25OHD to 1,25 dihydroxyvitamin D, regardless of the amount of 25OHD available. Another probable reason is that a minor or weak effect of 25OHD on hypocalcemia has been covered by other confounders such as bilateral CCND and parathyroid antotransplantation, although these factors were not significant on the multivariate analysis.

In our study, we also evaluated whether preoperative VDD reduces the accuracy of postoperative PTH in predicting post-thyroidectomy hypocalcemia. The results showed the accuracy of postoperative PTH as a predictor for hypocalcemia appeared similar between patients with 25OHD < 20 and≥ 20 ng/mL (Table 3 and Figure 1). This is consistent with the finding of Lang et al [15] and Cherian et al [16] studies. However, there are some different features between their studies and ours. Lang et al [15] defined VDD as 25OHD level <15ng/mL. Cherian et al [16] study included considerably variable extent of surgery; with only 18.6% patients underwent TT plus CCND. In contrast, other authors reported that postoperative serum PTH had lower accuracy in predicting hypocalcemia in patients with VDD when compared to patients without VDD. Sam et al [20] demonstrated that the area under the ROC curve for postoperative serum PTH to predict hypocalcemia in patients without VDD was 0.93 (95% CI: 0.86 –1.00; *P* = 0.0001), but that of patients with VDD was 0.68 (95% CI: 0.39–0.97; *P* = 0.23). It should be noted that the sample size is relatively small in that study; the total sample size is 74, and only 16 patients suffered VDD. The smaller sample size may affect the accuracy of the results. Another study [19] also revealed that postoperative serum PTH was not a reliable predictor for hypocalcemia in patients with VDD based on a series of 203 cases. In their cases, the preoperative PTH level in VDD group was significant higher than that of control group (mean: 60.35 vs. 22.4 pg/mL, *P* = 0.0001), which suggested the presence of secondary hyperparathyroidism. The secondary hyperparathyroidism would influence the postoperative PTH level in VDD patients. However, the preoperative PTH level had no significant difference in patients with or without VDD (mean: 5.78 vs. 5.23pmol/L, *P* = 0.072) in our study. Thus, the secondary hyperparathyroidism may explain the discrepancy between aforementioned study and ours.

It is worth noting that several limitations were present. First, although the study included a relatively homogeneous population of thyroid cancer patients and the operations were performed by a single surgeon, the sample size remains relatively small; our findings might have been purely the result of the underpowering of the study. However, based on a previous study [30], 57 subjects in each group (VDD and VDS) were enough to detect a difference of 15% in hypocalcemia using Flemming's model with a type 1 error (α) of 0.05 and a power (β) of 80%. Second, although there was a high incidence of VDD in our series, the number of patients with severe VDD (<10 ng/mL) was small (only 23 patients). Whether severe VDD increases the risk of hypocalcemia need to be further studied. Third, the surgical volume of the surgeon was significantly associated with the postoperative hypocalcemia of thyroid surgery [31–33]. Therefore, in order to eliminate the impact of the individual surgeon experience on postoperative complications, we only included the patients operated by a single experienced thyroid surgeon (ZH. Li), but which was a potential limitation for universality.

In conclusion, although VDD was common in our cohort, it was not a significant risk factor for hypocalcemia following TT+CCND, and did not affect the accuracy of postoperative PTH as a predictor of post-thyroidectomy hypocalcemia. Thus, routine preoperative screening for vitamin D seems to be unnecessary.

## MATERIALS AND METHODS

### Patients

After approval of the Institutional Research and Ethical Board, we retrospectively analyzed the data of consecutive thyroid cancer patients undergoing TT with CCND by a single surgeon (Li ZH) in the department of thyroid and breast surgery of West China Hospital between October 2015 and April 2016. All patients who were more than 18 years old and pathologically confirmed papillary thyroid cancer were included. Patients with evidence of hyperthyroidism, concomitant parathyroid disease, metabolic bone disease, liver or renal dysfunction and patients with previous thyroid surgery were excluded. Patients with abnormal value of preoperative serum calcium, PTH, albumin or creatinine were also excluded from the study.

### Methods

Surgical techniques have been described previously [10, 34]. Briefly, TT was performed using a capsular dissection technique with routine identification and preservation of the recurrent laryngeal nerves and parathyroid glands. Devascularized or inadvertently removed parathyroid glands were chopped into 1mm^3^ fragments and autotransplanted into the non-tumor side sternocleidomastoid muscle. Intraoperative nerve monitoring and nano-carbon suspension were used to help identifying and preserving the recurrent laryngeal nerve and parathyroid glands in all patients. All patients underwent TT plus unilateral or bilateral CCND with or without modified radical neck dissection (MRND).

Preoperative vitamin D, serum calcium, albumin and PTH levels were measured within one week before surgery in all patients. Postoperative vitamin D, serum calcium and PTH levels were determined at each morning for three days after surgery. Serum calcium concentration was adjusted for serum albumin: adjusted calcium = 0.8*(4.0-serum albumin) + serum calcium. Hypocalcemia was defined as adjusted serum calcium < 2.0 mmol/L with or without any clinical symptoms or signs of hypocalcemia, including numbness and paresthesias of the fingertips, toes, and perioral area, positive Chvostek's or Trousseau's sign, and tetany, which is a frequently used definition [2, 7, 29, 35]. All patients with asymptomatic hypocalcemia were treated with oral calcium (calcium carbonate 1.2-2.4 g/d). Symptomatic hypocalcemia was managed with parenteral calcium and an oral 1,25-dihydroxy vitamin D3 (calcitriol) supplementation of 0.5–1.0 g/d. Similar to other studies [11, 16, 28, 29], VDD was defined as serum 25-hydroxyvitamin D (25OHD) levels <20 ng/mL in our study. 25OHD levels ≥20 ng/mL defined as vitamin D sufficiency (VDS). No prophylactic oral calcium or vitamin D supplementation was administrated in the preoperative period. All the patients were divided into two groups (VDD and VDS). Reference ranges of biochemical parameters were 2.10–2.60 mmol/L for serum calcium, 1.60-6.90 pmol/L for serum PTH, and 47.7-144 nmol/L for serum 25-OHD concentrations (to convert to ng/mL, divide by 2.5) in our hospital.

### Statistical analysis

Continuous variables data was presented as mean ± standard deviation (SD). Categorical data was presented as frequency along with percentages. Independent sample *t*-test and Mann–Whitney *U* test were used to compare continuous variables based on the data distribution, while Chi-square test or Fisher's exact test was used for categorical variables. To identify independent predictor of postoperative hypocalcemia, linear logistic regression analysis was performed. Receiver operator characteristic curve (ROC) was constructed and the area under the ROC was used to measure the predictive accuracy and compared between both groups. A *P* value < 0.05 was considered to be statistically significant. All analyses were performed using the SPSS 22.0 (SPSS, Inc., an IBM Company, Chicago, IL, USA) and Stata version 11.0 (Stata Corporation, College Station, TX, USA).
